# Targeting *MET* Amplification as a New Oncogenic Driver

**DOI:** 10.3390/cancers6031540

**Published:** 2014-07-22

**Authors:** Hisato Kawakami, Isamu Okamoto, Wataru Okamoto, Junko Tanizaki, Kazuhiko Nakagawa, Kazuto Nishio

**Affiliations:** 1 Department of Medical Oncology, Kinki University Faculty of Medicine, 377-2 Ohno-higashi, Osaka-Sayama, Osaka 589-8511, Japan; E-Mails: kawakami_h@dotd.med.kindai.ac.jp (H.K.); wokamoto@east.ncc.go.jp (W.O.); Junko_Tanizaki@dfci.harvard.edu (J.T.); nakagawa@med.kindai.ac.jp (K.N.); 2 Center for Clinical and Translational Research, Kyushu University Hospital, 3-1-1 Maidashi, Higashiku, Fukuoka 812-8582, Japan; 3 Division of Transrlational Research, Exploratory Oncology Research & Clinical Trial Center, National Cancer Center, 6-5-1 Kashiwanoha, Kashiwa, Chiba 277-8577, Japan; 4 Lowe Center for Thoracic Oncology, Dana-Farber Cancer Institute, HIM223, 450 Brookline Avenue, Boston, MA 02215, USA; 5 Department of Genome Biology, Kinki University Faculty of Medicine, 377-2 Ohno-higashi, Osaka-Sayama, Osaka 589-8511, Japan; E-Mail: knishio@med.kindai.ac.jp

**Keywords:** MET, gene amplification, non-small cell lung cancer, gastric cancer, fluorescence in situ hybridization (FISH), polymerase chain reaction (PCR), crizotinib

## Abstract

Certain genetically defined cancers are dependent on a single overactive oncogene for their proliferation and survival, a phenomenon known as “oncogene addiction”. A new generation of drugs that selectively target such “driver oncogenes” manifests a clinical efficacy greater than that of conventional chemotherapy in appropriate genetically defined patients. *MET* is a proto-oncogene that encodes a receptor tyrosine kinase, and aberrant activation of MET signaling occurs in a subset of advanced cancers as result of various genetic alterations including gene amplification, polysomy, and gene mutation. Our preclinical studies have shown that inhibition of MET signaling either with the small-molecule MET inhibitor crizotinib or by RNA interference targeted to MET mRNA resulted in marked antitumor effects in cancer cell lines with *MET* amplification both *in vitro* and *in vivo*. Furthermore, patients with non-small cell lung cancer or gastric cancer positive for *MET* amplification have shown a pronounced clinical response to crizotinib. Accumulating preclinical and clinical evidence thus suggests that *MET* amplification is an “oncogenic driver” and therefore a valid target for treatment. However, the prevalence of *MET* amplification has not been fully determined, possibly in part because of the difficulty in evaluating gene amplification. In this review, we provide a rationale for targeting this genetic alteration in cancer therapy.

## 1. Introduction

Certain genetically defined cancers are dependent on a single overactive oncogene for their proliferation and survival, a phenomenon known as “oncogene addiction” that is exemplified by the *BCR-ABL* fusion gene in chronic myeloid leukemia as well as by mutant forms of the epidermal growth factor receptor (EGFR) gene and by the *EML4-ALK* fusion gene in non-small cell lung cancer (NSCLC). A new generation of drugs that selectively target such “driver oncogenes” and which include tyrosine kinase inhibitors (TKIs) has shown a therapeutic efficacy greater than that of conventional chemotherapy in individuals with these specific molecular alterations [1,2]. The identification of additional kinase oncogenes would thus be expected to facilitate the development of new molecularly targeted therapies.

The proto-oncogene *MET* encodes the receptor tyrosine kinase c-MET (or MET). The binding of its ligand—the hepatocyte growth factor (HGF)—to MET results in tyrosine phosphorylation of the receptor and activation of downstream signaling pathways mediated by phosphoinositide 3-kinase (PI3K) and AKT, by signal transducer and activator of transcription 3 (STAT3), or by RAS and mitogen-activated protein kinase (MAPK). Whereas normal activation of MET is essential for wound healing and embryonic development [3,4], excessive activation of MET signaling in a subset of advanced cancers [5,6,7,8,9] results in the up-regulation of cell proliferation, motility, migration, and invasion [3,10]. Although such aberrant MET signaling potentially arises from genetic alteration or dysregulation of *MET* [11], the target potential of *MET* alterations including polysomy, gene amplification, and gene mutation has not been well established.

## 2. Preclinical Findings

To investigate the biological impact of *MET* amplification or mutation, we have examined the effects of a MET-TKI and of a small interfering RNA (siRNA) specific for MET mRNA on cell survival and signal transduction in NSCLC cells with or without such genetic alterations of *MET* [12]. Several types of *MET* mutation, including those that affect the kinase domain or other domains of the encoded protein, have been identified in tumors. The small-molecule drug crizotinib (PF-02341066) inhibits the tyrosine kinase activity of MET as well as that of oncogenic fusion variants of anaplastic lymphoma kinase (ALK) [13,14]. We found that inhibition of MET signaling with crizotinib or MET siRNA induced apoptosis that was accompanied by attenuation of the phosphorylation (activation) of AKT and the MAPK extracellular signal-regulated kinase (ERK) in NSCLC cells with *MET* amplification but not in those positive for a non-kinase domain mutation (N375S or deletion of exon 14) of *MET* [12]. These results suggest that MET signaling is essential for the survival of NSCLC cells with *MET* amplification but not for that of those without this genetic alteration, including those with a non-kinase domain mutation of *MET*, although MET-TKIs have been shown to be active against MET with mutations in the kinase domain [15]. Crizotinib also showed a marked antitumor effect on lung cancer xenografts positive for *MET* amplification, whereas it had little effect on those negative for *MET* amplification, including those with a *MET* mutation, consistent with our results obtained *in vitro*. Together, these findings suggest that gene amplification, but not gene mutation, renders *MET* active as a driver oncogene.

In gastric cancer, in which gain-of-function mutations of *MET* are exceedingly rare [16,17,18], activation of *MET* has been attributed to gene amplification [19,20,21]. A highly selective MET-TKI, PHA-665752, was shown to have potential antitumor efficacy in gastric cancer cells with *MET* amplification [22]. We therefore also examined the potential antitumor action of crizotinib or MET siRNA in gastric cancer cells positive or negative for *MET* amplification [23]. Consistent with our results obtained with NSCLC cells [12], we found that inhibition of MET signaling by either of these agents resulted in induction of apoptosis associated with inhibition of AKT and ERK phosphorylation in gastric cancer cells with *MET* amplification but not in those without it, suggesting that MET signaling is essential for the survival of *MET* amplification-positive cells. Crizotinib also manifested a marked antitumor effect on gastric cancer xenografts positive for *MET* amplification, whereas it had little effect on those negative for this genetic change. Crizotinib thus showed a pronounced antitumor action both *in vitro* and *in vivo* specifically in gastric cancer cells positive for *MET* amplification.

In summary, our preclinical studies have shown that gene amplification, but not gene mutation, confers “oncogenic driver” potential on *MET*. Tumor cells positive for *MET* amplification are thus dependent on (“addicted to”) sustained MET activity for their growth and survival, with the result that inhibition of MET signaling either with a small-molecule MET inhibitor or by RNA interference targeted to MET mRNA has marked antitumor effects both *in vitro* and *in vivo*. These findings provide a rationale for targeting *MET* amplification with MET-TKIs in the clinical setting.

## 3. Prevalence of *MET* Amplification in Cancer Patients

**Figure 1:**
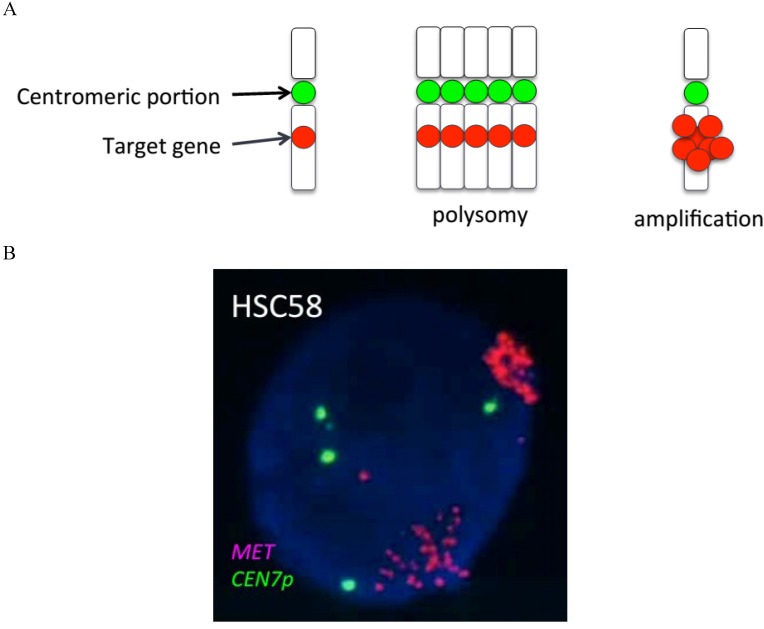
(**A**) Schematic comparison of gene amplification and polysomy. The ratio of the copy number for the target gene to that for the centromeric portion of the chromosome distinguishes an increased copy number of the target gene attributable to gene amplification from that resulting from extra copies of the chromosome (polysomy). (**B**) FISH analysis of a gastric cancer cell line (HSC58) positive for *MET* amplification. The image shows a single cancer cell, with green and red signals corresponding to *CEP7* (*CEN7p*) and the *MET* locus, respectively.

Given the potential of MET-targeted therapy for cancer with *MET* amplification, it is important to determine the prevalence of this gene alteration in patients with advanced cancer. Unfortunately, however, different studies have used different methods and criteria to detect *MET* amplification (Table 1 and Table 2). Studies based on fluorescence *in situ* hybridization (FISH) analysis have identified *MET* amplification in up to ~5% of patients with NSCLC [24,25,26,27] or gastric cancer [20,28,29], whereas an increase in *MET* copy number was found in up to ~20% of NSCLC [30,31,32,33,34,35] and gastric cancer [36,37,38,39,40] patients by Southern blot analysis or with a polymerase chain reaction (PCR)-based assay. To understand the reason for this discrepancy, it is important to recognize the difference between the two genetic mechanisms—gene amplification and polysomy—that can give rise to an increase in gene copy number in malignant tumors. Gene amplification is defined as a copy number increase for a specific gene (or group of genes) on a given chromosome arm without a change in copy number for genes located in other regions of the chromosome [41]. On the other hand, polysomy gives rise to a copy number gain for a given gene as a result of the presence of extra copies of the entire chromosome. Of note, polysomy for chromosome 7 (the chromosome on which *MET* is located) was indeed observed ~30% of NSCLC [27] and gastric [29] tumors with an increased *MET* copy number. Furthermore, such tumors might not be MET driven, given that breast tumors with an increased copy number for the human epidermal growth factor receptor 2 (HER2) gene as a result of polysomy 17 behave as HER2-negative tumors [42]. Southern blot analysis and PCR-based assays identify a gain in gene copy number regardless of the underlying cause and are thus unable to discriminate gene amplification from polysomy (Figure 1A). This methodological limitation is sometimes overlooked in determination of the prevalence of *MET* amplification in cancer. 

**Table 1:** Prevalence of *MET* amplification and increased *MET* gene copy number (GCN) in NSCLC.

Study	Number of Patients	Technique	Classification	Positivity (%)
Camidge *et al*. (2010) [43]	66	FISH	*MET/CEP7* ratio > 2.0	0
Onozato *et al*. (2009) [33]	148	PCR based	GCN > 2	1.4
Kubo *et al*. (2009) [34]	100	PCR based	GCN > 5	2.0
Bean *et al*. (2007) [30]	16	PCR based	GCN > 5	3.0
Go *et al*. (2010) [27]	180	FISH	*MET/CEP7* ratio > 2.0	3.9
Okamoto *et al*. (2014) [44]	229	FISH	*MET/CEP7* ratio > 2.2	3.9
Cappuzzo *et al*. (2009) [45]	447	FISH	*MET/CEP7* ratio > 2.0	4.1
Onitsuka *et al*. (2010) [32]	183	PCR based	GCN > 1.31	4.4
Okuda *et al*. (2008) [31]	213	PCR based	GCN > 3	5.6
Beau-Faller *et al*. (2008) [35]	106	PCR based	GCN > mean + 2SD of 30 normal lung DNA samples	20.8

FISH, fluorescence in situ hybridization; PCR, polymerase chain reaction; GCN, gene copy number; CEP7, centromeric portion of chromosome 7.

**Table 2:** Prevalence of *MET* amplification and increased *MET* gene copy number (GCN) in gastric cancer.

Study	Number of Patients	Technique	Classification	Positivity (%)
Janjigian *et al*. (2011) [29]	38	FISH	*MET/CEP7* ratio > 2.0	0
Kawakami *et al*. (2013) [46]	266	FISH	*MET/CEP7* ratio > 2.2	1.5
Lennerz *et al*. (2011) [28]	267 (junctional and gastric)	FISH	*MET/CEP7* ratio > 2.2	2.2
Hara *et al*. (1998) [20]	154	FISH	NA	3.9
Liu *et al*. (2014) [47]	196	FISH	*MET/CEP7* ratio > 2.0	6.1
Graziano *et al*. (2011) [40]	216	PCR based	GCN ≥ 5	9.7
Tsugawa *et al*. (1998) [21]	70	Slot blot analysis	Ratio > 2 (relative to normal mucosa)	10.0
Nakajima *et al*. (1999) [19]	128	Southern blot analysis	Ratio > 2 (relative to normal mucosa)	10.2
Lee *et al*. (2011) [39]	472	PCR based	GCN ≥ 4	21.2
Shi *et al*. (2012) [48]	128	PCR based	GCN ≥ 4	30.5

FISH, fluorescence in situ hybridization; PCR, polymerase chain reaction; GCN, gene copy number; CEP7, centromeric portion of chromosome 7; NA, not available.

On the other hand, FISH analysis is a semiquantitative method that can be performed with two probes for determination of the number of signals both for a target gene and for the centromeric portion of the corresponding chromosome. Given that the number of centromeric signals directly indicates the copy number of the chromosome, FISH analysis reveals the copy number increase for the target gene from the ratio of the copy number of the gene to that of the chromosome (Figure 1). Comparative genomic hybridization (CGH) is another molecular cytogenetic approach to the identification of gene amplification. CGH analyzes copy number variation for whole chromosomes or subchromosomal regions relative to ploidy level in the DNA of a test sample in comparison with a reference sample [49]. Although CGH has proved to be an efficient and reproducible technique, it remains relatively expensive to perform and requires a well-equipped laboratory and a high level of operator expertise.

FISH is thus currently the gold standard for detection of gene amplification. According to the recent ASCO/CAP guidelines for *HER2* testing, gene amplification is defined as positive with a target gene/centromere ratio of >2.2, negative with a ratio of <1.8, and equivocal with a ratio between 1.8 and 2.2 [50]. Importantly, polysomy, which is mechanistically distinct from gene amplification, is mostly associated with a ratio in the equivocal range [51].

With the strict definition of *MET* amplification as a *MET/CEP7* (centromeric region of chromosome 7) ratio of >2.2 as determined by FISH analysis, we identified nine out of 229 patients with advanced NSCLC (3.9%) as being positive for *MET* amplification [44]. We also found that four out of 266 gastric cancer patients (1.5%) were positive for *MET* amplification as determined with a combination of PCR-based screening and FISH confirmation [46]. These results suggest that *MET* amplification identifies a small but clinically important subgroup of cancer patients who are likely to respond to MET-TKIs.

## 4. Clinical Response to Crizotinib in *MET* Amplification—Positive Cancer Patients

To date, at least 17 MET-TKIs with kinase selectivity profiles ranging from highly selective to multitargeted have been or are currently being subjected to clinical evaluation [52]. Although several agents including cabozantinib [53] and foretinib [54] have made good progress, they are multitargeted MET-TKIs, and so little is known of the relation between their efficacy and *MET* amplification. In NSCLC, *MET* amplification is one of the mechanisms responsible for the development of resistance to EGFR-TKIs, with dual inhibition of EGFR and MET having been shown to induce apoptosis in such resistant cells [55]. Combination treatment with an EGFR-TKI and tivantinib, a selective MET-TKI with microtubule-disrupting activity similar to that of vincristine [56], has been evaluated in clinical trials, but the efficacy of this approach remains unclear. Among the MET-TKIs examined, however, crizotinib has consistently shown efficacy in patients with cancer positive for *MET* amplification.

Preliminary reports of the clinical response of patients with *MET* amplification-positive cancer to crizotinib have come from an enriched molecular cohort of individuals with advanced cancer in a phase I trial of this drug (A8081001, ClinicalTrials.gov identifier NCT00585195). This cohort includes patients with various tumor types harboring specific genetic alterations of *MET* or *ALK*, including *MET* amplification defined as a *MET/CEP7* ratio of >2.2 (but not polysomy 7, kinase domain-activating mutations of *MET*, or other chromosomal translocations leading to altered transcriptional regulation of *MET*) as well as *ALK* chromosomal translocation or gene amplification. A patient with stage IV lung adenocarcinoma that was negative for *ALK* rearrangement but positive for high-level *MET* amplification (*MET/CEP7* ratio of >5.0) started treatment with crizotinib at a dose of 250 mg twice a day [57]. The patient achieved a maximum reduction in aggregate tumor measurement of 54.8% after 4 months of such therapy and thereafter continued the study treatment showing a partial response. A patient with *MET* amplification-positive glioblastoma was also treated with crizotinib at 250 mg twice a day [58]. After 2 months of treatment, the first scheduled cranial magnetic resonance imaging (MRI) scan revealed a 40% reduction in tumor size, and after 4 months a restaging cranial MRI examination confirmed this effect to be stable. Administration of crizotinib was continued for a total of 6 months, until the patient manifested disease progression.

Another study revealed a pronounced clinical response to crizotinib in two of four patients with gastric cancer positive for *MET* amplification (*MET/CEP7* ratio of >2.2) [28]. After 1 week of crizotinib treatment, one patient experienced a rapid symptomatic response with an increase in appetite, reduction in pain, and improvement in performance status. A computed tomography (CT) scan at the end of treatment cycle 2 (8 weeks) revealed a partial tumor response, which was confirmed at 12 weeks. Another patient also showed rapid clinical improvement, with reduced pain and improved performance status, after 1 week of crizotinib treatment. Time to progression for these two patients on crizotinib treatment was ~112 and 105 days, respectively.

Crizotinib was approved by the U.S. Food and Drug Administration for the treatment of *ALK* rearrangement-positive NSCLC in 2011, and a recent report has addressed the clinical efficacy of this agent in a clinical practice setting [59]. A male patient with stage IV squamous cell lung cancer was found to be positive for *MET* amplification (*MET/CEP7* ratio of >2.2) and negative for *ALK* rearrangement by FISH analysis. He was treated with crizotinib monotherapy at the normal dose of 250 mg twice daily. An almost complete response of tumors in the left lung and a major response of the primary tumor to therapy were demonstrated by chest CT and positron emission tomography (PET)-CT after 8 weeks of therapy.

Preliminary results of the NCT00585195 phase I study for patients with *MET* amplification-positive NSCLC were reported at the 2014 Annual Meeting of the American Society of Clinical Oncology (ASCO) [60]. Patients were categorized into three classes according to *MET* amplification status as determined by FISH analysis: low (*MET/CEP7* ratio of ≥1.8 to ≤2.2), intermediate (*MET/CEP7* ratio of >2.2 to <5.0), and high (*MET/CEP7* ratio of ≥5.0). Thirteen patients with a low (*n* = 1), intermediate (*n* = 6), or high (*n* = 6) *MET/CEP7* ratio received crizotinib. Of the 12 evaluable patients, four (33%) showed a partial response and were found to have an intermediate (*n* = 1) or high (*n* = 3) *MET/CEP7* ratio. These findings are thus suggestive of an association between the *MET/CEP7* ratio and the clinical benefit of crizotinib in patients with *MET* amplification-positive cancer.

The accumulating clinical evidence thus suggests that *MET* amplification as strictly defined by a *MET/CEP7* ratio of >2.2 has the potential to act as an oncogenic driver and thereby to render at least a subset of affected tumors responsive to MET-TKIs such as crizotinib. Not all *MET* amplification-positive cancer patients respond to MET-TKI treatment, however, and most such patients who do respond, even those who show an initial marked response, eventually develop resistance to MET-TKIs. Preexisting and acquired resistance to MET-TKIs is thus an important clinical problem that is shared with other targeted therapies. Several mechanisms of resistance to MET-TKIs have been identified in preclinical models, including additional mutations in the activation loop of MET [61], ligand-dependent activation of EGFR signaling [61,62], *SND1-BRAF* fusion [63], and amplification and overexpression of wild-type *KRAS* [64]. Further characterization of such mechanisms will be important to provide a basis for the development of effective therapies for patients with MET-TKI resistance.

## 5. Conclusions

*MET* amplification has been identified as a potential oncogenic driver for several neoplasms, and targeted therapy with MET-TKIs for such tumors is thus a reasonable and effective treatment. Clinical trials of such drugs are strongly warranted for patients with advanced malignancies positive for *MET* amplification as strictly defined by a *MET/CEP7* ratio of >2.2 determined by FISH.
